# C/EBP*β* Isoforms Expression in the Rat Brain during the Estrous Cycle

**DOI:** 10.1155/2015/674915

**Published:** 2015-04-29

**Authors:** Valeria Hansberg-Pastor, Ana Gabriela Piña-Medina, Aliesha González-Arenas, Ignacio Camacho-Arroyo

**Affiliations:** ^1^Departamento de Biología, Facultad de Química, Universidad Nacional Autónoma de México, DF, Mexico; ^2^Unidad de Investigación en Reproducción Humana, Instituto Nacional de Perinatología-Facultad de Química, Universidad Nacional Autónoma de México, Ciudad Universitaria, Avenida Universidad 3000, Coyoacán, 04510 Ciudad de México, DF, Mexico; ^3^Departamento de Medicina Genómica y Toxicología Ambiental, Instituto de Investigaciones Biomédicas, Universidad Nacional Autónoma de México, DF, Mexico

## Abstract

The CCAAT/enhancer-binding protein beta (C/EBP*β*) is a transcription factor expressed in different areas of the brain that regulates the expression of several genes involved in cell differentiation and proliferation. This protein has three isoforms (LAP1, LAP2, and LIP) with different transcription activation potential. The role of female sex hormones in the expression pattern of C/EBP*β* isoforms in the rat brain has not yet been described. In this study we demonstrate by western blot that the expression of the three C/EBP*β* isoforms changes in different brain areas during the estrous cycle. In the cerebellum, LAP2 content diminished on diestrus and proestrus and LIP content diminished on proestrus and estrus days. In the prefrontal cortex, LIP content was higher on proestrus and estrus days. In the hippocampus, LAP isoforms presented a switch on diestrus day, since LAP1 content was the highest while that of LAP2 was the lowest. The LAP2 isoform was the most abundant one in all the three brain areas. The LAP/LIP ratio changed throughout the cycle and was tissue specific. These results suggest that C/EBP*β* isoforms expression changes in a tissue-specific manner in the rat brain due to the changes in sex steroid hormone levels presented during the estrous cycle.

## 1. Introduction

The CCAAT/enhancer-binding proteins (C/EBP) is a family of transcription factors that consist of six members (C/EBP*α*-C/EBP*ζ*) named according to their chronological order of discovery. These proteins are solely eukaryotic and bind as dimers to specific DNA sequences to regulate gene transcription. They have a highly conserved C-terminal bZIP domain comprising a leucine-zipper dimerization domain and a basic DNA binding region. Particularly, the isotype C/EBP*β* is involved in different functions such as cell proliferation and differentiation, cell survival, apoptosis, metabolism, and immune response [1, 2].

The expression of C/EBP*β* is regulated by a number of factors like hormones, nutrients, cytokines, mitogens, and several transcription factors (CREB, NF*κ*B, Sp1, and STAT-3) [2, 3]. C/EBP*β* has three isoforms that are translated from a single transcript by the alternative use of different AUG initiation codons within the same open reading frame [4, 5]. C/EBP*β* isoforms were first identified in the liver and therefore known as LAP1 and LAP2 (for liver activating proteins) and LIP (for liver inhibitory protein). LAP2 (34 kDa) is suggested to be a stronger transactivator than the full-length isoform LAP1 (38 kDa). The shorter isoform LIP (20 kDa) lacks the N-terminal transactivation domains and frequently acts as a dominant negative [1, 6]. However, the transactivation potential of these isoforms depends on the LAP/LIP ratio, which is important to modulate cell fate.

C/EBP*β* has been associated with key functions in the central nervous system (CNS) such as learning, memory, and cognition [2]. It is widely expressed in several brain regions, both in neurons and astrocytes [7, 8]. In the neonatal male rat brain, C/EBP*β* is found in the cerebellum, cerebral cortex, hippocampus, thalamus, and brainstem. In mice neuroblastoma N2A cells, C/EBP*β* participates in neurite extension and cell differentiation through the activation of PI3K signaling [9]. In the dentate gyrus of the hippocampus, C/EBP*β* is important for the proliferation of newborn cells. Mice lacking this protein have reduced newborn cell survival, decreased neuronal differentiation, and fewer cells proliferating in the subgranular zone of the dentate gyrus [10]. Also, C/EBP*β* has been associated with protection of cerebellar granular neuron death [11] or as part of the neuronal injury response to activate regeneration-associated genes [12]. Despite the important role of C/EBP*β* in the CNS and the different transcriptional activity of its isoforms, most studies report its expression without considering the three isoforms, usually reporting only the abundant LAP2 isoform.

Sex steroid hormones regulate a number of different processes affecting not only reproductive traits but also the CNS. Estradiol (E2) and progesterone (P4) participate in memory consolidation, cognitive functions, brain plasticity, neuronal damage protection, and brain tumors growth [13–16]. These hormones regulate these functions by modulating the expression of target genes through the interaction with its intracellular receptors [17–19]. The regulation of C/EBP*β* expression by hormones has been studied in other sex hormone target organs such as endometrium and mammary gland. C/EBP*β* is an essential factor during embryo implantation and decidualization in mice and primates [20–22]. Studies with knockout mice for C/EBP*β* show that the animals are infertile due to failure in ovulation and luteinization [23]. C/EBP*β* is also important for E2-induced proliferation of uterine epithelial cells in nonpregnant mice [20] and for the normal development and function of the mammary gland [24]. LIP isoform expression increases in the mammary gland during rat pregnancy and after parturition [25]. There is evidence that changes in C/EBP*β* isoform ratio (LAP/LIP) are important for the cellular response to ovarian P4 in the reproductive tract [26]. C/EBP*β* can also interact with estrogen receptor (ER) to induce the expression of genes involved in milk production in the mammary gland [27]. Some recent evidence shows that C/EBP*β* binds to progesterone receptor (PR) intron 2 in human uterine stromal cells, probably modulating its expression [28].

Notwithstanding the effects of sex hormones and C/EBP*β* in different reproductive organs and the CNS, there are no studies regarding the effects of sex hormones in C/EBP*β* expression in the brain. In this study we demonstrated that the expression of the three C/EBP*β* isoforms in different brain areas depends on sex hormone level variations presented throughout the estrous cycle.

## 2. Materials and Methods

### 2.1. Animals

24 intact Sprague Dawley female rats (90 days of age, 250 g) certified by Harlan Laboratories, Inc. (Harlan, Mexico City, MEX) were maintained under a 12 h light-dark cycle (lights on from 6:00 am to 6:00 pm) with food and water available* ad libitum*. Rats, which presented at least three regular 4-day estrous cycles, were used as determined by daily vaginal smears. Rats were killed by decapitation in the morning (10:00 am) of metestrus, diestrus, proestrus, and estrus. The brains were dissected into three regions according to the Atlas of Paxinos and Watson [29]: prefrontal cortex, hippocampus, and cerebellum. All samples were immediately processed for protein extraction. The experiments were performed according to the Official Mexican Norm (NOM-062-ZOO-1999) in strict accordance with the recommendations of the Guide for the Care and Use of Laboratory Animals of the National Institutes of Health of the USA.

### 2.2. Estrous Cycle Evaluation

Daily, vaginal smears were stained to determine the cycle phases of the rats. The slides with the smears were first stained with a ready-to-use hematoxylin (Biocare Medical, CA, USA) for 10 min and gently washed with tap water. Slides were dipped in a saturated lithium carbonate solution for 3 min to intensify the staining, washed with water to remove the salt, and air dried at room temperature (RT). Then, the slides were covered with alcoholic eosin (Biocare Medical, CA, USA) for 10 min, washed with 70% ethanol, and air dried at RT. The vaginal smears were observed under an optical microscope Olympus BX41 (Olympus, PA, USA).

### 2.3. Western Blot

Samples were homogenized in RIPA lysis buffer with protease inhibitors (1 mM EDTA, 2 *μ*g/mL leupeptin, 2 *μ*g/mL aprotinin, 1 mM PMSF) and proteins were obtained by centrifugation at 12500 rpm, at 4°C for 15 min and quantified using a NanoDrop 2000 Spectrophotometer (Thermo Scientific, MA, USA). 70 *μ*g of total protein was separated by electrophoresis on a 12% SDS-PAGE at 20 mA; colored markers (Bio-Rad, CA, USA) were included for size determination. Gels were transferred to nitrocellulose membranes (Millipore, MA, USA) (35 mA) in semidry conditions at RT for 1 h. Membranes were blocked with 3% nonfat dry milk and 1% bovine serum albumin at RT for 2 h and then incubated with an antibody against the three C/EBP*β* isoforms (0.6 *μ*g/mL) (ab32358, Abcam, Cambridge, ENG) at 4°C for 48 h. Afterwards, blots were incubated with anti-rabbit secondary antibody (1 : 7500) conjugated to horseradish peroxidase (Santa Cruz Biotechnology, TX, USA) at RT for 45 min. In order to correct for differences in the amount of total protein loaded in each lane, C/EBP*β* isoforms content was normalized to that of *α*-tubulin. Blots were stripped with glycine (0.1 M, pH 2.5, 0.5% SDS) at RT for 30 min and incubated with 0.2 *μ*g/mL of mouse anti-*α*-tubulin monoclonal antibody (sc-5286, Santa Cruz Biotechnology, TX, USA) at 4°C overnight. Blots were incubated with a 1 : 3000 dilution of goat anti-mouse IgG conjugated to horseradish peroxidase (Santa Cruz Biotechnology, TX, USA) at RT for 45 min. Chemiluminescence signals were detected exposing membranes to Kodak Biomax Light Film (Sigma-Aldrich, MO, USA) using Supersignal West Femto as peroxidase substrate (Thermo Scientific, MA, USA) with a constant exposure time of 5 min for C/EBP*β* and 30 seconds for *α*-tubulin. The antigen-antibody complex was detected as the area under a peak corresponding to a band density (the area is given in inches with a default scale of 72 pixels/inch) in a semiquantitative way using a 14.1 megapixels digital Canon camera (SD1400IS, Canon, Mexico City, MEX) and the ImageJ 1.45S software (National Institutes of Health, USA). In order to minimize interassay variations, all western blots were carried out in parallel for each brain region.

### 2.4. Statistical Analysis

All data were analyzed and plotted using the GraphPad Prism 5.0 software for Windows 8.1 (GraphPad Software, CA, USA). A statistical analysis between comparable groups was performed using a two-way ANOVA with a Bonferroni posttest. The LAP/LIP ratio for each brain region was analyzed using a Kruskal-Wallis test followed by a Dunn posttest. A value of *P* < 0.05 was considered statistically significant as stated in the figure legends.

## 3. Results

The three isoforms of C/EBP*β*, LAP1 (38 kDa), LAP2 (34 kDa), and LIP (20 kDa) were clearly identified by western blot in the cerebellum, prefrontal cortex, and hippocampus of the rat. In all the studied brain areas the 34 kDa LAP2 isoform was the more abundant one.

**Figure 1:**
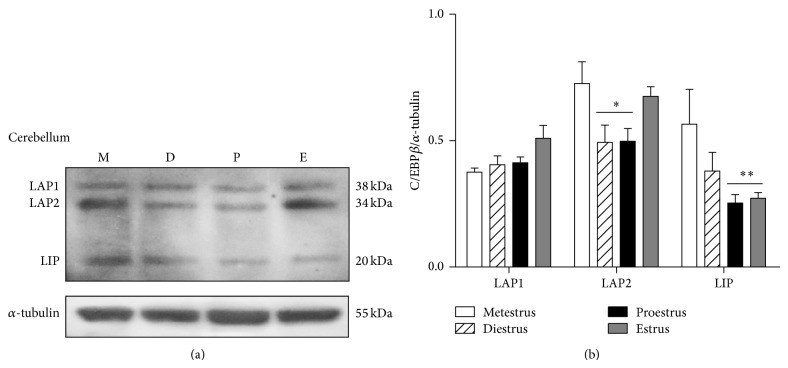
C/EBP*β* isoforms LAP1, LAP2, and LIP content in the rat cerebellum throughout the estrous cycle. (a) A representative western blot for the three C/EBP*β* isoforms during metestrus (M), diestrus (D), proestrus (P), and estrus (E). (b) Densitometric analysis of C/EBP*β* isoforms with respect to *α*-tubulin content. The data represent the mean ± S.E.M, *n* = 6, ^∗^ *P* < 0.05 LAP2 diestrus and proestrus versus metestrus and estrus; ^∗∗^ *P* < 0.05 LIP proestrus and estrus versus metestrus and diestrus.

**Figure 2:**
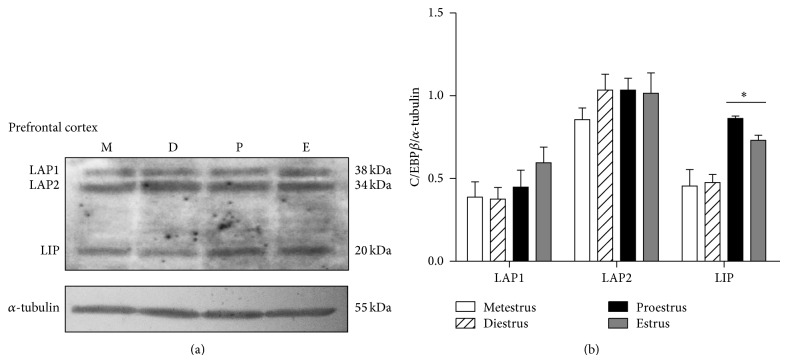
C/EBP*β* isoforms content in the rat prefrontal cortex throughout the estrous cycle. (a) A representative western blot for LAP1, LAP2, and LIP isoforms content during metestrus (M), diestrus (D), proestrus (P), and estrus (E). (b) Densitometric analysis of LAP1, LAP2, and LIP content relative to that of *α*-tubulin. The data represent the mean ± S.E.M, *n* = 6, ^∗^ *P* < 0.05 LIP proestrus and estrus versus metestrus and diestrus.

**Figure 3:**
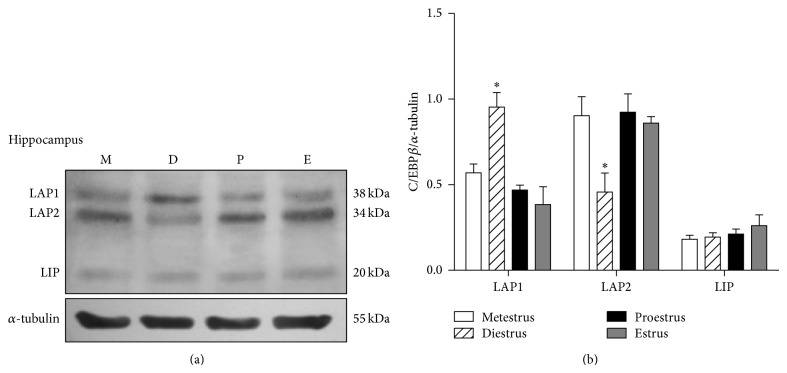
C/EBP*β* isoforms content in the rat hippocampus along the estrous cycle. (a) A representative western blot for the three isoforms during metestrus (M), diestrus (D), proestrus (P), and estrus (E). (b) Densitometric analysis of LAP1, LAP2, and LIP content relative to that of *α*-tubulin. The data represent the mean ± S.E.M, *n* = 6, ^∗^ *P* < 0.05 LAP1 and LAP2 diestrus versus all the other days.

In the cerebellum, the content of LAP1 showed a nonsignificant increase on estrus day. LAP2 content diminished on diestrus and proestrus days, while LIP showed a reduced content during proestrus and estrus (Figure 1). In the prefrontal cortex, LIP was the unique isoform that presented changes throughout the estrous cycle. This isoform increased its content during proestrus and estrus (Figure 2). In the hippocampus, the larger isoforms LAP1 and LAP2 showed an inverse expression on diestrus day since LAP1 content increased in this day while that of LAP2 diminished. The shorter isoform LIP did not change its content along the estrous cycle (Figure 3).

The LAP/LIP ratio changes throughout the estrous cycle in a tissue specific manner. In the cerebellum the LAP/LIP ratio increased throughout the estrous cycle from metestrus to estrus (*P* < 0.023 metestrus versus estrus), while in the prefrontal cortex the LAP/LIP ratio decreased during proestrus (*P* < 0.029 diestrus versus proestrus) and then moderately increased on estrus. In the hippocampus, the LAP/LIP ratio slightly diminished during proestrus and estrus, but no statistical significant changes were observed (Table 1).

## 4. Discussion

This work demonstrates that C/EBP*β* is expressed in different areas of the rat brain and changes its content throughout the estrous cycle. The three C/EBP*β* isoforms LAP1, LAP2, and LIP were detected in the cerebellum, prefrontal cortex, and hippocampus. Cortés-Canteli and coworkers [9] previously reported the expression of C/EBP*β* in all these regions in the male neonatal rat, but without studying each isoform.

Changes in sex steroid hormone levels throughout the estrous cycle influence brain function and morphology [14, 30–32]. E2 and P4 show a specific concentration pattern throughout the estrous cycle. E2 levels begin to increase during the late diestrus and show a maximum peak during the morning of proestrus. The increase in estrogen levels is followed by a rise in P4 levels during mid to late proestrus and the early estrus [33, 34]. The fluctuations in C/EBP*β* isoforms content may depend on changes in E2 and P4 levels and the expression of sex hormone receptors.

In the cerebellum the decrease in LAP2 content during diestrus and proestrus could be due to the increase in E2 levels while the decrease in LIP isoform during proestrus and estrus could be related to the increase in both E2 and P4. The increase in LIP isoform content in the prefrontal cortex could also be induced by E2 and P4. The LAP isoforms change in hippocampus during diestrus could be due to the increasing levels in E2 that precede the high hormone levels observed during proestrus. Many of the effects of sex hormones depend on the actions of ER and PR that modulate target gene expression. These receptors are widely expressed in different brain areas including the cortex, hippocampus, hypothalamus, and cerebellum [35–37]. There is no evidence that C/EBP*β* is directly regulated by ER and PR, but a microarray study shows that PR can induce C/EBP*β* expression in breast cancer cells [38]. Nonetheless, more studies are needed using ovariectomized animals and receptor antagonists in order to confirm the direct effect of sex hormones in C/EBP*β* expression.

In addition to a transcriptional regulation, sex hormones could influence C/EBP*β* isoforms translation, given that they are translated from a single mRNA [39]. Different signal transduction pathways regulate the function of the translation initiation factors eIF2 and eIF4E, which determine the ratio of C/EBP*β* isoforms [5]. There is evidence that E2 causes polyribosomes to accumulate in the dendrites of hippocampal neurons suggesting mRNA translation regulation [40]. In rat primary neuronal cultures of hippocampal and cortical regions, E2 increases phosphorylation of ribosomal protein S6 and eIF4E binding protein 1 (4EBP1) through the activation of ERK, and this promotes an increase in dendritic mRNA translation [41]. These studies suggest a possible role of gonadal sex hormones in C/EBP*β* isoform translation.

Sex hormones modulate the animal behavior through changing the structure and function of different brain areas. Besides mating behavior, female animals show alterations in anxiety, learning, and memory and in the response to stress depending on the estrous cycle phase [34, 42]. E2 and P4 can modulate hippocampal and cortical functions in the rat influencing learning and memory processes [43–45]. High E2 levels during proestrus enhance hippocampal memory consolidation, while in diestrus the animals show impairment in learning and memory [46, 47]. There is evidence that C/EBP*β* expression in the hippocampus is associated with the consolidation of new memories [48–50]. In our study we observed a change in LAP1/2 isoform expression during diestrus in the hippocampus suggesting a possible role of these isoforms in memory consolidation.

In different cell models, the isoforms ratio is important to determine cell fate and variations in the LAP/LIP ratio can significantly activate or inhibit expression of target genes [51, 52]. The LAP/LIP ratio is therefore an important indicator of C/EBP*β* transcriptional activity [53]. In rat white adipose tissue, a caloric restriction reduced the LAP/LIP ratio, which was associated with cell differentiation [54]. In the hepatic glucose metabolism, hyperglycemia increased the LAP/LIP ratio, which in turn promoted an increase in genes associated with gluconeogenesis [55]. Until now there are no data available regarding the hormone regulation of the LAP/LIP ratios in the brain. In the cerebellum, the isoform ratio increases along the estrous cycle suggesting a key role of the LAP isoforms in this brain region and particularly during estrus. In contrast, the low LAP/LIP ratio during metestrus suggests an important function for the LIP isoform. In the prefrontal cortex, the decrease in the LAP/LIP ratio during proestrus suggests an important role of the LIP isoform in regulating gene expression when sex steroid hormone levels are high. Given that this isoform is usually considered as a dominant negative, its increase could downregulate different target genes. In the hippocampus the LAP/LIP ratio appears to decrease from metestrus to estrus, but given the variations in the data no significant changes were observed. However, more studies are needed to understand the actions of C/EBP*β* isoforms in the brain and the role of sex hormone receptors in the regulation of such actions.

## 5. Conclusions

This work is the first to describe the expression of the three C/EBP*β* isoforms in the brain and its changes under physiological conditions during the estrous cycle of the rat. The C/EBP*β* isoforms expression in the cerebellum, prefrontal cortex, and hippocampus may be regulated by E2 and P4. The LAP and LIP isoforms expression changes throughout the estrous cycle and is tissue-specific. Our work shows important changes in the expression of C/EBP*β* isoforms during the estrous cycle that might be relevant to the female reproductive adaptation.

## Figures and Tables

**Table 1:** The LAP/LIP ratio in the different brain areas throughout the estrous cycle. The ratio was expressed as the average of both LAP1 and LAP2 isoforms content to that of the LIP isoform. The data represent the ratios in each estrous cycle phase (metestrus, diestrus, proestrus, and estrus) in the different brain areas (cerebellum, prefrontal cortex, and hippocampus) with their respective standard deviation (SD).

	Cerebellum	Prefrontal cortex	Hippocampus
	Ratio ± SD	Ratio ± SD	Ratio ± SD
Metestrus	1.9 ± 0.62^∗^	2.7 ± 0.54	8.1 ± 1.75
Diestrus	2.4 ± 0.71	3.0 ± 0.95	7.3 ± 2.90
Proestrus	3.6 ± 0.83	1.7 ± 0.35^∗∗^	6.6 ± 1.52
Estrus	4.4 ± 0.91	2.2 ± 0.34	4.8 ± 1.27

^∗^ *P* < 0.023 metestrus versus estrus, ^∗∗^ *P* < 0.029 diestrus versus proestrus.
